# The effect of neuromuscular electrical stimulation on serum glucose levels in children and adolescents with type-1 diabetes mellitus: a single group clinical trial

**DOI:** 10.1186/s12902-022-01149-6

**Published:** 2022-10-11

**Authors:** Fatemeh Fallah, Morteza Alijanpour, Soraya Khafri, Mohammad Pournasrollah, Ghadam Ali Talebi

**Affiliations:** 1 Department of Physiotherapy, Babol University of Medical Sciences, Babol, Iran; 2 Department of Pediatrics, Non-Communicable Pediatric Disease Research Center, Babol University of Medical Sciences, Babol, Iran; 3 Social Determinates of Health Research Center, Health Research Institute, Babol University of Medical Sciences, Babol, Iran; 4 The Clinical Research Development Unit of Amirkola Children’s Hospital, Babol University of Medical Sciences, Babol, Iran; 5 Mobility Impairment Research Center, Babol University of Medical Sciences, Babol, Iran

**Keywords:** Diabetes mellitus, Type1- electric stimulation- blood glucose

## Abstract

**Background:**

This study aimed to evaluate the effect of Neuromuscular Electrical Stimulation (NMES) on serum glucose level in children and adolescents with type-1 diabetes.

**Methods:**

This before-after, single-group, clinical trial was conducted on 29 patients with type-1 diabetes mellitus with the age range of 7–18 years. The patients underwent NMES in two 20-minute phases on the quadriceps and hamstrings muscles, three sessions per week for a period of 8 weeks. Fasting Blood Sugar (FBS), measured in two ways, by glucometer and laboratory testing, was considered as the primary outcome and the glycated hemoglobin (HbA1c) and the total daily dose (TDD) of insulin were measured as the secondary outcomes. The laboratory FBS and HbA1c were measured 1 day before the intervention (as a baseline value) and then 2 and 6 weeks after the last session of intervention. FBS by glucometer and total daily dose of insulin were recorded daily from 2 weeks before the intervention to the last day of the intervention and consequently, the weekly average of these variables was calculated and used for statistical analysis.

**Results:**

The serum level of FBS (measured by glucometer) and the total daily dose of insulin reduced significantly 2 weeks after beginning of intervention. The laboratory serum level of FBS decreased significantly in the second week after the end of intervention compared to the baseline values. Although the HbA1c level decreased at follow-up period (2 and 6 weeks after the intervention), it was not significant.

**Conclusion:**

It seems that 8 weeks of NMES has beneficial effects on the reduction of FBS and TDD of insulin therefore, it could be suggested as the contributory treatment in management of children and adolescents with type-1 diabetes.

**Trial registration:**

The study was registered at https://fa.irct.ir/user/trial/51739/view (IRCT20100523003998N1) in date of 25/10/2020.

## Background

Type-1 Diabetes Mellitus (T1DM) is one of the most common endocrine and metabolic diseases in childhood in which insulin therapy is considered as a life-saving and life-long solution [1]. Children with T1DM may show symptoms such as polyuria, polydipsia, polyphagia, weight loss, fatigue and blurred vision, which usually appear several days to a few weeks before the diagnosis [2]. Due to the complications of this disease and its costs, prevention and treatment are absolutely vital. Insulin therapy is the preferred treatment for T1DM, but HbA1c control, lifestyle changes and exercise are also recommended [3]. The American Diabetes Association guideline recommends at least 1 h of moderate to vigorous daily physical activity for children with T1DM. Herbst A and et al. in a cohort study in pediatrics with T1DM, demonstrated that regular physical activity can lead to better control of blood glucose, including a lower HbA1c level, and in female patients, lower BMI as well and the frequency of physical activity (number of repetitions per week) was reported as an important factor in blood glucose control [4]. These researchers mentioned that increasing the frequency of exercise does not advance the risk of severe hypoglycemia or hypoglycemia with loss of consciousness or seizure [4]. Fear of hypoglycemia during and after exercise is probably the main barrier to physical activity in children and adolescents with T1DM [5]. Meanwhile, these patients may sometimes have to refrain from physical activity due to other physical problems such as musculoskeletal injuries, fractures and dislocations or other diseases and hospitalization. In such circumstances, using of an alternative form of exercise is recommended, since regular uninterrupted physical activity is needful for these patients. In this regard, Neuromuscular Electrical Stimulation (NMES) can be a complementary method of exercise in patients with diabetes that creates muscle contractions. NMES involves the application of electrical stimulation to the peripheral nervous system in order to create muscle contraction [6]. A previous study of NMES application for healthy individuals has shown that the body’s overall glucose uptake can be increased significantly during and after NMES [7]. It is also possible to lower postprandial blood glucose levels by applying 30 minutes of NMES to the lower extremities [8]. In recent years, many studies have been performed to evaluate the effects of NMES in adults with type-2 diabetes, almost all of which have confirmed the positive effects of NMES on glycemic control in these patients. Nevertheless, no studies have yet reported the effects of NMES on children and adolescents with T1DM. Moreover, most long-term studies have been conducted using portable devices without therapist’s supervision. Therefore, we decided to investigate the possible effects of NMES on diabetes control factors such as FBS, total daily dose of insulin and HbA1c in children and adolescents with T1DM who were aged seven to 18 years old.

## Methods

### Study design

**Fig. 1:**
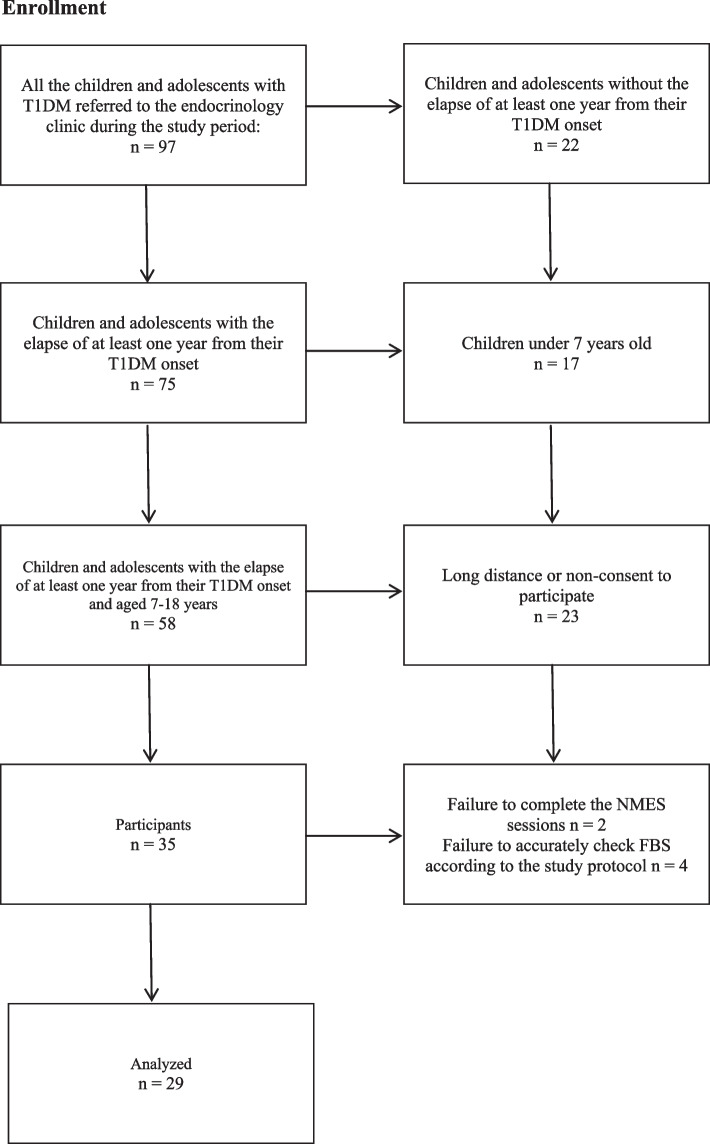
Flowchart of the study protocol

This before-after, single-group, clinical trial was conducted at the Amirkola Children’s Hospital in Babol, Iran from September 2020 to February 2021. Considering the decrease of 1.5 units of FBS after the intervention compared to before [9], the effect size of 0.52 at the confidence level of 95%, and the power of 80%, the sample size was determined to be 32. Consequently, with the assumption of 10% drop, 35 children and adolescents were meant to be included in the study, which were later reduced to 29 during the study with the permission of the University Research Council due to the critical COVID-19 conditions. The data of 29 patients were thus statistically analyzed (Fig. 1). All the patients were insulin-dependent and used one of its conventional types (NPH or regular) or the Basal bolus protocol.

All the patients and their parents completed informed consent forms to participate in the study. The patients’ routine diet and medications did not change during the intervention. They continued their normal activities during the intervention period and there was no change in their activity level. All the participants were physically examined by a pediatric endocrinologist as well. Their weight was measured with a digital (Balas) scale made in Iran and their height with a stadiometer. Their Body Mass Index (BMI) was calculated as weight (kg) divided by height (m^2^).

Data recording forms including daily information about the FBS levels (checked by the glucometer) and the daily insulin intake were distributed among the patients or their parents to be filled daily. The FBS and HbA1c were measured in the laboratory before the intervention and then 2 and 6 weeks after the last intervention session (follow-up). As to make the results better interpreted, FBS (as measured by the glucometer) and the daily dose of insulin intake were also recorded 2 weeks before the intervention. All the patients had at least a one-year history of T1DM and all of them already knew how to measure FBS properly with a glucometer and the correct way to inject insulin. In addition, they had already been taught how to measure the amount of carbohydrate of the foods and the appropriate amount of total daily dose of insulin (TDD) at the hospital. Furthermore, during the intervention period, all the patients were examined by specialist physician every other week as to control the blood glucose and total daily dose of insulin changes. This study was approved by the Ethics Committee of Babol University of Medical Sciences with a code no: (IR.MUBABOL.REC.1399.298), and also registered at the Iranian Registry of Clinical Trials (IRCT) with the number (IRCT20100523003998N1).

### Inclusion and exclusion criteria

The inclusion criteria were the elapse of at least 1 year from the onset of T1DM and age range of 7–18 years and no history of known neuromuscular diseases, seizures, and use of anticonvulsant drugs, heart diseases, recent infectious diseases, thyroid disorders, hypercalcemia and sensory impairment. None of the subjects could be professional athletes either. The exclusion criteria were the patient’s non-consent to participate in the study, failure to complete the NMES sessions, failure to accurately check their fasting blood sugar according to the study protocol, and demonstrating intolerance to electrical stimulation during the study.

### Intervention

The patients were told to eat a light meal (a carbohydrate-rich snack such as fruits) 1 h before the intervention [10]. Their blood glucose level was checked using a glucometer, 10 minutes before the intervention to avoid possible hypoglycemia or hyperglycemia. The intervention was performed from 2 to 6 pm at the physiotherapy department of Amirkola Children’s Hospital. A 4-channel stimulator (710P PLUS, Novin Medical Engineering Co., made in Iran) was used for all the patients to elicit muscle contraction. The stimulation protocol for each NMES session consisted of a symmetrical, biphasic, balanced square pulse of a frequency of 35 Hz, pulse duration of 300 microseconds, with 5 seconds on and 15 seconds off process. The intensity was set at a level with which the patients were comfortable. The protocol included two 20-minute phases with an interval of 10 minutes between the two stages to prevent fatigue and possible discomfort specifically in the children. In the first step, four 6 × 4 cm electrodes were placed bilaterally on the right and left bulk of the quadriceps muscles [11]. The second stage was similar to the first one, but in this stage the electrodes were placed on the left and right bulk of the hamstring muscles. The patients laid in the supine position while stimulating the quadriceps and in the prone position while stimulating the hamstring and isometric muscle contractions were induced. All the sessions were performed under a physiotherapist’s supervision. The intervention was performed three times a week (every other day) and lasted overally 8 weeks.

### Outcomes

#### Primary outcome

##### Fasting Blood Sugar (FBS)

Fasting blood sugar levels (mg/dl) were measured in two ways: first, by the patients or their parents with a glucometer (Glucocard 01, made in Japan). They recorded the FBS level daily, beginning from 2 weeks pre-intervention until the last intervention session (totally 10 weeks). The weekly average of fasting blood sugar was calculated and used for statistical analysis. The second way of measurement was by the laboratory testing (glucose oxidase method), which was performed three times including before the intervention and then 2 and 6 weeks after the last intervention session. In this method the blood serum was first isolated by centrifuge, and then 1 mL of blood was used with a laboratory kit (Pars Azmun Company, Iran). All these measurements were performed at the same time (8 am) and at the same laboratory for all the patients while fasting (for 8 h).

#### Secondary outcomes

##### Total daily dose of insulin

The patients’ daily insulin intake dose from 2 weeks before the intervention to the last day of the intervention (totally 10 weeks) was recorded in the daily information form and its weekly average was calculated and used for statistical analysis.

##### HbA1c level

HbA1c levels (%) were measured in the blood samples by a laboratory specialist before the intervention and then 2 and 6 weeks after the last session using a Diazyme kit (made in the US) by taking 2 cc of blood for the enzymatic method.

The measured values for all of the variables in pre-intervention period were defined as baseline values.

### Data analysis

For the statistical analysis of the data SPSS 18 statistical software was applied and Shapiro-Wilk’s test was used to evaluate the normality of the data distribution. In addition, Repeated-Measures ANOVA was used to measure and compare the variables at different times. The researchers applied the effect size to express the effect of the intervention on the response variable (ηp2). And finally the Bonferroni-corrected test was used to compare the measurements on the different occasions.

The level of statistical significance was set at 0.05 for all the tests and Pearson’s correlation test was considered as a proper test in order to examine the relationship between BMI and changes in laboratory FBS, HbA1c, weekly average of FBS, and weekly average of TDD.

## Results

**Fig. 2:**
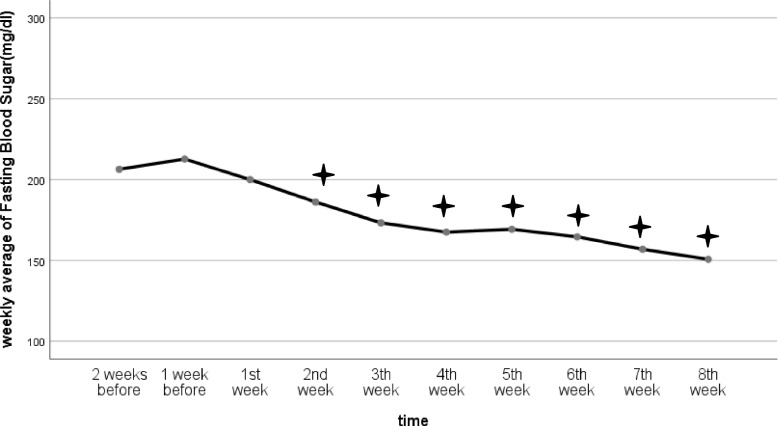
Changes in weekly average of fasting blood sugar levels. The ✦ shows significant changes at each time compared to before intervention

As mentioned before, a total of 29 children and adolescents with T1DM who had been referred to the endocrine clinic of Amirkola Children’s Hospital, including 18 girls (62.1%) and 11 boys (37.9%) in the age range of seven to 18 years old participated in this study. The measured baseline characteristics included age (13.20 ± 3.22 years), height (152.06 ± 16.10 cm), weight (46.24 ± 15.24 kg), and body mass index (19.31 ± 3.93 kg/cm^2^). All the samples participated in all the NMES sessions. Based on ANOVA test, the FBS level (measured by a glucometer) was decreased significantly from the second through 8 week of intervention compared to before intervention values (F:16.68, df:3.71, P:0.001, η_p_^2^:0.37) (Table 1, Fig. 2).

**Table 1:** ANOVA test for the FBS level (measured by the glucometer) and Weekly Average of TDD

Variables	FBS (mg/dl) Mean ± SD	TDD Mean ± SD
Time		
2 weeks before	206.40 ± 49.27	36.72 ± 18.52
1 week before	212.72 ± 54.58	36.37 ± 17.76
1 week after	200.02 ± 52.33	35.47 ± 18.57
2 weeks after	186.13 ± 51.95	34.10 ± 18.52
3 weeks after	173.30 ± 53.89	33.54 ± 18.38
4 weeks after	167.57 ± 42.68	33.10 ± 15.71
5 weeks after	169.32 ± 42.93	33.66 ± 16.39
6 weeks after	164.67 ± 39.45	33.49 ± 15.47
7 weeks after	156.93 ± 34.73	34.04 ± 17.07
8 weeks after	150.75 ± 36.75	33.55 ± 17.17
*P*. Value	0.001^*^	0.003^*^

*FBS* Fasting Blood Sugar, *TDD* Total Daily Dose of insulin intake

*indicates a statistically significant finding

**Fig. 3:**
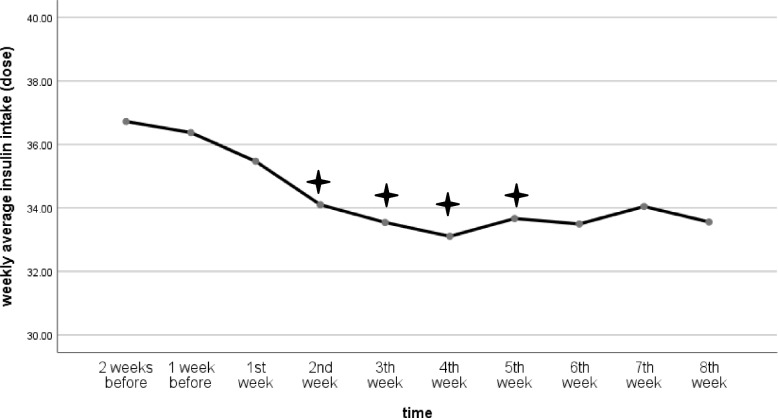
Changes in the weekly average of daily dose of insulin. The ✦ shows significant changes at each time compared to before intervention

The total daily dose of insulin was also reduced significantly from second through fifth week of intervention compared to the before intervention dose. (F:4.68, df:3.35, P:0.003, η_p_^2^:0.14). After the fifth week of intervention, the total daily dose of insulin showed no significant changes compared to baseline values (Table 1, Fig. 3).

Based on the laboratory measurements, the serum level of FBS was decreased significantly in the second follow-up week (2 weeks after the completion of intervention) with respect to baseline values (F:6.31, df:2, P:0.003, η_p_^2^:0.18); but the level of FBS in the sixth follow-up week was not significant relative to baseline values (Table 2).

**Table 2:** ANOVA test for laboratory FBS and HbA1c

Variables	FBS (mg/dl) Mean ± SD	HbA1c (%) Mean ± SD
Time		
Before	202.82 ± 55.71	10.01 ± 2.13
2 weeks after	163.26 ± 54.31	9.67 ± 2.01
6 weeks after	197.06 ± 70.06	9.53 ± 1.82
*P*. Value	0.003^*^	0.256

*FBS* Fasting Blood Sugar, *HbA1c* glycated hemoglobin

*indicates a statistically significant finding

The HbA1c levels decreased at follow-up weeks (2 and 6 weeks after the end of intervention period) with respect to baseline values, but this change was not statistically significant (F:1.36, df:2, P:0.265, η_p_^2^:0.05), (Table 2).

Pearson’s correlation test was used to evaluate the relationship between BMI and laboratory FBS differences, HbA1c differences, FBS differences, and differences in the total daily dose of insulin. As Table 3 illustrates, there was no significant correlation between BMI and differences in laboratory FBS, HbA1c and the weekly average of FBS (*P* > 0.05 for all the variables). Nonetheless, the results of Pearson’s correlation test showed that there was an inverse, moderate correlation between BMI and the weekly average differences in the total daily dose of insulin (P: 0.006, r: 0.497).

**Table 3:** The correlation between BMI and differences in laboratory FBS, HbA1c, FBS by glucometer, and TDD

Variables		laboratory FBS	HbA1c	FBS by glucometer	TDD of insulin
BMI	r	0.33	0.13	0.21	−0.49
	*P*-Value	0.07	0.48	0.25	0.00^*^

*FBS* Fasting Blood Sugar, *HbA1c* glycated hemoglobin, *TDD* Total Daily Dose

*indicates a statistically significant finding

## Discussion

In this study, 8 weeks of Neuromuscular Electrical Stimulation (NMES) significantly reduced FBS levels and the total daily dose of insulin intake in children and adolescents with T1DM. HbA1c levels also decreased in these patients, although not significantly. According to these findings, NMES can lead to reduction of the FBS. To the researchers’ knowledge, there are no studies that have reported the effects of NMES on children and adolescents with type-I diabetes mellitus or generally in patients with type-1 diabetes. Nonetheless, some previous studies have showed that the NMES can reduce the blood glucose in healthy individuals and patients with type-2 diabetes [10, 12–15]. Based on the present study, the NMES has similar effects on blood glucose in patients with type-1 diabetes. The findings showed that the laboratory FBS reduced significantly at the second follow-up week (2 weeks after the last intervention session) compared to baseline values. However, the amount of laboratory FBS increased at the sixth follow-up week relative to second follow-up week and a significant difference was not detected between the sixth follow-up week and the baseline values. Based on this finding it can be concluded that it is essential to continue the NMES protocol to maintain and enforce its desired effects. In 2003, Taku Hamda et al. conducted the first study on the effect of a single low-frequency electrical stimulation session on human glucose metabolism and found that the stimulatory effect of electrical stimulation on whole body glucose uptake persisted not only during the stimulation but also for at least 90 minutes after it [13]. Georges Jabboure et al. showed that serum concentrations of blood glucose measured in patients with type-2 diabetes 60 and 120 min after NMES dropped significantly compared to the control group [12]. Since NMES elicits muscle contractions, its mechanism of action is probably similar to that of exercise. Exercise can increase blood glucose uptake by two mechanisms: 1.Insulin-independent mechanism and glucose uptake through muscle contraction, and 2.Insulin sensitivity increase, which will lead to the transfer of insulin-dependent Glut4 to the cell membrane [13]. Moreover, unlike the orderly recruitment of motor units in voluntary contraction, during electrical stimulation, the motor units related to type-2 fibers are more active due to their thicker axons and lower resistance to electrical stimulation and thus result in more glycogen utilization [16]. AMP-activated protein kinase (AMPK) activation in response to increasing the ratio of AMP to ATP in the muscle cells can have positive effects on glucose uptake and the oxidation of fatty acids in the skeletal muscles [10]. Another possible mechanism associated with the increased glucose intake during electrical stimulation is that during this process tissue blood flow increases [17].

To our knowledge, the present study is the first study to investigate the effect of NMES on insulin levels in patients with T1DM in which the results demonstrated that 8 weeks of NMES can reduce the total daily dose of insulin. Since no studies have been conducted on the effects of NMES on insulin levels in diabetic patients, it is hard to discuss the mechanisms of the effects of NMES on this variable. Nevertheless, since NMES involves the application of electrical stimulation to the peripheral nervous system to elicit muscle contraction [6], it seems that the NMES uses a mechanism similar to exercise and brings about the similar effects. Studies on T1DM have consistently proved that physical activity is associated with a reduced need for insulin. This decrease has been reported to vary from 6% [18] to more than 15% [19]. A meta-analysis and systematic review conducted to evaluate the clinical outcomes of exercise in patients with T1DM showed that exercise can improve some markers of T1DM such as BMI, peak VO_2_ and LDL in adults, and insulin dose, waist circumference, LDL and triglycerides in children [20]. It has been shown that one to three sessions of exercise per week can reduce the dose of insulin in adolescents with T1DM [21]. Researches postulated that the fear of hypoglycemia after exercise may be a possible cause for the patients to reduce their insulin dose [21]. In addition, as another possible mechanism, the exercise can reduce the blood Glucose levels, however, the reduction of insulin dose depends on the intensity and duration of exercise and the insulin levels [22].

In the present study, the HbA1c level reduced following 8 weeks of NMES, but not significantly. However, the results of previous studies on the effect of NMES on HbA1c levels in patients with type-2 diabetes were contradictory [9, 15, 23, 24]. Louis Crow et al. showed that 8 weeks of electrical stimulation reduced HbA1c levels significantly in men with type-2 diabetes [23]. Nevertheless, the contradictory result was reported by Oonagh M. Giggins et al., that eight-week electrical stimulation did not lead to significant changes in HbA1c levels in patients with type-2 diabetes [9]. In another study, the findings indicated that 12 weeks of NMES in patients with type-2 diabetes and cerebral palsy reduced the levels of HbA1c, total cholesterol and LDL significantly [24]. These conflicting results may be due to the differences in the electrical stimulation parameters applied and the duration of the intervention or the sample size. It has been reported that physical activity can improve the blood glucose levels in short term and HbA1c levels in long term in patients with type-2 diabetes; consequently, more than 8 weeks of physical activity is needed to reduce HbA1c levels significantly [25]. The HbA1c level is a much better indicator than blood glucose or urine glucose levels, and indicates the average blood glucose level in the last 8–12 weeks (depending on the individual) [26]. In the present study, the number of stimulated muscles, intensity of stimulation, frequency of stimulation per week, and total duration of the intervention, were not sufficient to induce significant changes in HbA1c levels.

According to the result of the present study, children and adolescents with T1DM who were overweight and obese responded less to electrical stimulation than children and adolescents with T1DM with normal weight, since the improvement in insulin levels was lower in the obese and overweight children. These results emphasize the importance of weight control and the prevention of obesity and overweight in children and adolescents with T1DM.

This study had several limitations and also has some implications for future studies:

The present study was a single-group study with no control group for comparison and the sample size was small. At first we tried to include 35 participants in the study but later on due to covid pandemic the number was inevitably reduced to 29. However, the results obtained from the final power showed that the results of this study can be considered as reliable (power = 91%). In addition, the intervention period was probably not sufficient to demonstrate a significant change in HbA1c levels. For better results, it is recommended to conduct a study with a larger sample size, longer intervention period (more than 8 weeks), and a control group. On the other hand, drawing a comparison between the NMES group and a routine exercise group can also help determine if NMES can truly be considered as a good alternative to exercise. Due to ethical and financial issues and the COVID-19 pandemic, the laboratory FBS was not measured immediately after the last session of intervention, we measured laboratory FBS and HbA1c two and 6 weeks after the last session. The NMES was performed between 2 p.m. and 6 p.m. in this study. It would have been better if the blood glucose level had been measured 2 hours after NMES in addition to the fasting blood glucose to have more accurate results.

## Conclusion

According to the results, 8 weeks of neuromuscular electrical stimulation can reduce FBS and the total daily dose of insulin. The positive effects of electrical stimulation on blood glucose and insulin intake suggest that this method can be used as a complementary tool and in some cases even as an alternative to exercise, especially during periods when the patient is unable to perform physical activity.

## Data Availability

The datasets used and/or analyzed during the current study are available from the corresponding author on reasonable request.

## Abbreviations

NMES: Neuromuscular electrical stimulation
T1DM: Type 1 diabetes mellitus
FBS: Fasting blood sugar
TDD: Total daily dose
HbA1c: Glycated hemoglobin
